# The Ahmed shunt versus the Baerveldt shunt for refractory glaucoma: a meta-analysis

**DOI:** 10.1186/s12886-016-0265-6

**Published:** 2016-06-08

**Authors:** Shiming Wang, Xiaoming Gao, Nana Qian

**Affiliations:** Ningbo Aier Guangming Eye Hospital, 8 Huancheng north Road, Ningbo, 315020 China

**Keywords:** Ahmed glaucoma valve implant, Baerveldt implant, Refractory glaucoma

## Abstract

**Background:**

The purpose of this study was to compare the efficacy and tolerability of the Ahmed glaucoma valve (AGV) implant and the Baerveldt implant for the treatment of refractory glaucoma.

**Methods:**

We comprehensively searched four databases, including PubMed, EMBASE, Web of Science, and the Cochrane Library databases, selecting the relevant studies. The continuous variables, namely, intraocular pressure reduction (IOPR) and a reduction in glaucoma medication, were pooled by the weighted mean differences (WMDs), and the dichotomous outcomes, including success rates and tolerability estimates, were pooled by the odds ratio (ORs).

**Results:**

A total of 929 patients from six studies were included. The WMDs of the IOPR between the AGV implant and the Baerveldt implant were 1.58 [95 % confidence interval (CI): −2.99 to 6.15] at 6 months, −1.01 (95 % CI: −3.40 to 1.98) at 12 months, −0.54 (95 % CI: −4.89 to 3.82) at 24 months, and −0.47 (95 % CI: −3.29 to 2.35) at 36 months. No significant difference was detected between the two groups at any point in time. The pooled ORs comparing the AGV implant with the Baerveldt implant were 0.51 (95 % CI: 0.33 to 0.80) for the complete success rate and 0.67 (95 % CI: 0.50 to 0.91) for qualified success rate. The Baerveldt implant was associated with a reduction in glaucoma medication at −0.51 (95 % CI: −0.90 to −0.12). There were no significant differences between the AGV implant and the Baerveldt implant on the rates of adverse events.

**Conclusions:**

The Baerveldt implant is more effective in both its surgical success rate and reducing glaucoma medication, but it is comparable to the AGV implant in lowering IOP. Both implants may have comparable incidences of adverse events.

## Background

Glaucoma drainage devices have been widely used in the management of refractory glaucoma because of a high risk for failure (e.g., failed trabeculectomy, neovascular and uveitic glaucoma, and traumatic glaucoma) with standard filtration surgery [1, 2]. This includes eyes that have ever undergone eye surgery that caused scarring of the conjunctiva, such as trabeculectomy [3], and the presence of secondary glaucoma that are known to have poor success rates with trabeculectomy, such as neovascular glaucoma and traumatic glaucoma [4]. Despite the high-risk profile of these patients, aqueous drainage devices as a first-line surgical treatment have had good levels of success [5, 6].

In the past two decades, several types of glaucoma drainage implants have been developed [7]. Two of the most commonly implanted aqueous drainage devices are the Ahmed glaucoma valve (AGV) and the Baerveldt implant. The AGV device incorporates a one-way valve to prevent postoperative hypotony and shallow anterior chambers [8, 9]. However, several studies reported that it was associated with high rates of encapsulation and inadequate intraocular pressure reduction (IOPR), often requiring postoperative glaucoma medications [10, 11]. The Baerveldt implants provide a greater surface area in the end plate for aqueous reabsorption, resulting in better IOP control, a reduction in glaucoma medication, and less encapsulation in the long term [12, 13].

Currently, a number of studies have compared these two types of glaucoma drainage implants in the treatment of refractory glaucoma [12–18]. As each implant has its own advantages and limitations, no definitive conclusions regarding objective outcome differences have been reached. Therefore, we performed a meta-analysis based on the literature to assess the whether the AGV or the Baerveldt implant has the predominant efficacy and safety in the treatment of refractory glaucoma.

## Methods

### Literature search

A comprehensive literature search of PubMed, ISI Web of Science, EMBASE, and the Cochrane library was performed to identify the relevant studies. Searches were conducted using the key words ‘glaucoma,’ ‘Ahmed,’ and ‘Baerveldt.’ We also searched Google Scholar and the websites of professional associations for relevant articles (http://www.aao.org; http://www.arvo.org). Once we identified the relevant articles, we searched their reference lists for additional articles. The final search was carried out in October 2015 without restrictions regarding methodology, publication year, or language.

### Inclusion and exclusion criteria

The inclusion criteria were: (i) study type: comparative studies; (ii) population: patients with refractory glaucoma (e.g., failed trabeculectomy, neovascular and uveitic glaucoma, and traumatic glaucoma); (iii) intervention: AGV implants versus Baerveldt implants; (iv) outcome variables: evaluation of at least one of the outcomes of interest mentioned below. The following exclusion criteria were used: (i) follow-up period less than 6 months; (ii) trials with a small sample size, (n <10); (iii) pediatric patient population; (iv) abstracts from conferences, full texts without raw data, duplicate publications, letters, and reviews. In the case of duplicate publications, the most recent series were included in this analysis.

### Outcome measures

The primary outcome was IOPR. The authors’ reported means and standard deviations (SD) of the IOPR were directly used. When these were not available, they were computed as follows: IOPR = IOP_baseline_ - IOP_endpoint_, SD_IOPR_ = (SD_baseline_^2^ + SD_endpoint_^2^ - SD_baseline_ × SD_endpoint_)^1/2^ [19]. The secondary outcome measure was the difference in reduction between glaucoma medications. For efficacy, the proportion of complete success and qualified success were also used. Complete success was defined as a target endpoint IOP (usually <21 mm Hg) without medication, and qualified success was defined as target endpoint IOP with or without medication. The third outcome was adverse event rates in either group.

### Data extraction

Two investigators (WSM and GXM) independently extracted the data from the selected studies. Data included first author, publication year, study design, location of the trial, follow-up, baseline patient characteristics, IOP, the number of glaucoma medications administered, and success rate. The numbers of withdrawals and patients reporting adverse events were also recorded. Disagreements were resolved through discussions and the achievement of consensus.

### Assessment of methodology quality

Two independent observers (WSM and GXM) assessed the quality of each study using a system intended for both randomized and nonrandomized studies [20]. This system was comprised of 27 items distributed among five subscales: reporting (10 items), external validity (three items), bias (seven items), confounding (six items), and power (one item). Disagreements about qualitative assessment were resolved through discussions, and a consensus was reached. The total score for each trial was expressed as a percentage of the maximum achievable score. Good quality refers to a quality score not lower than 50 %.

### Statistical analysis

The outcome measure was assessed on an intent-to-treat basis. The weighted mean differences (WMDs) with 95 % confidence intervals (CIs) and the odds ratios (ORs) with 95 % Cis were used to compare continuous and dichotomous variables, respectively. The random-effects model wasused to obtain a conservative estimate of the effect of different clinical characteristics among study groups and the variations in sample sizes among the studies [21]. Statistical heterogeneity between studies was evaluated using Cochran’s Q statistic and the *P*-value, and the I^2^ statistic was used to assess the quantity of heterogeneity. All statistical analysis was performed using Stata version 12.0 (Stata Corporation LP, College Station, TX, USA).

### Sensitivity analysis and publication bias

Sensitivity analysis was performed to evaluate the effect of the methodological characteristics of controlled clinical trials: trial design. To detect publication biases, the Begg and Egger measures were calculated [22, 23].

## Results

### Literature search

**Fig. 1:**
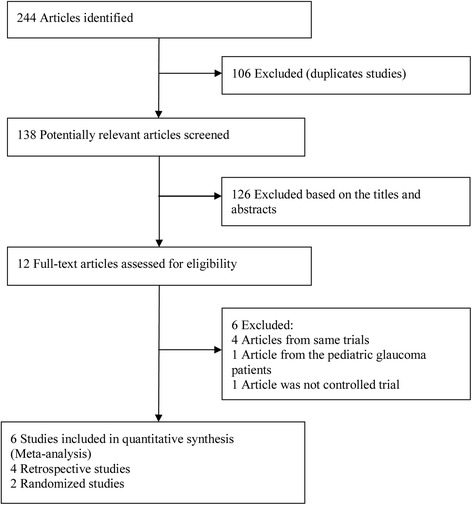
Flowchart of publication search and selection

The initial search yielded 244 relevant publications, of which 106 were excluded as duplicate studies, and 126 were excluded based on their titles and abstracts. The remaining 12 were retrieved for full-text review; four of them were excluded because they contained duplicated data [24–27]. One article compared AGV and Baerveldt implants in pediatric glaucoma [15], and another was not a controlled trial [28]. Ultimately, six studies published between 2004 and 2013 were included in the present meta-analysis [12–14, 16–18]. The trial selection process is presented in Fig. 1.

### Study characteristics and quality

Table 1 shows the main characteristics of the six included studies. These studies were conducted in various countries: four of them were from the United States, one was from Canada, and one was from Singapore. In total, 930 eyes from 929 patients (448 males and 481 females) were enrolled, with the mean age ranging from 48.1 to 69.2 years. The duration of the studies ranged from 12 to 48 months. Two trials had a prospective, parallel, randomized design, and four had a retrospective, nonrandomized design. All studies fulfilled the quality criteria (Downs and Blacks score) of being over 50 %. Table 1 displays the quality scores of the included studies.

**Table 1:** Characteristics of included studies

					Sex(male/female)				Quality score (%)
First Author (year)	Design	Location	No. eyes^a^	No. patients^a^	Ahmed	Baerveldt	Age (year)	Follow-up(mo)	
Syed et al. (2004) [18]	Retro	USA	32/32	32/32	12/20	19/13	61/58	12/12	59.37
Wang et al. (2004) [17]	Retro	Singapore	18/24	18/23	10/8	15/8	60/48.1	23.2/22.8	59.37
Tsai et al. (2006) [13]	Retro	USA	48/70	48/70	18/30	36/34	69.2/62.3	48/48	65.62
Goulet et al. (2008) [12]	Retro	USA	59/133	59/133	25/34	64/69	66.3/64.3	20/22.9	56.25
Budenz et al. (2011) [16]	RCT USA	143/133	143/133	73/70	70/63	65.4/62.2	12/12	78.12	
Christakis (2013) [14]	RCT	Canada	124/114	124/114	65/59	41/73	65/67	36/36	81.25

^a^Ahmed/Baerveldt

*Mo* months, *Retro* retrospective comparative study, *RCT* prospective randomized controlled

### IOPR and glaucoma medication reduction

Six studies compared the AGV implant with the Baerveldt implant in terms of the IOPR; five studies made comparisons at 6 months, six studies at 12 months, three studies at 24 months, and two studies made comparisons at 36 months. There was no statistically significant difference in the amount of IOP reduction between them during the intervals, with a WMD for the IOPR comparing the AGV implant with the Baerveldt implant of 1.58 (95 % CI:−2.99 to 6.15) at 6 months, −1.01 (95 % CI: −3.40 to 1.98) at 12 months, −0.54 (95 % CI: −4.89 to 3.82) at 24 months, and −0.47 (95 % CI: −3.29 to 2.35) at 36 months. At any point in time, substantial statistical heterogeneity was observed between the studies (Table 2). The studies were then divided into subgroups based on their design (retrospective and randomized). At any point in time, all of the subgroups showed no significant difference between the groups, with the exception of the randomized clinical trial (RCT) subgroup at 12 months (Table 2). Concerning glaucoma medication reduction, the Baerveldt implant achieved a greater change from baseline. The differences reached statistical significance, with a WMD comparing the AGV implant with the Baerveldt implant of −0.51 (95 % CI: −0.90 to −0.12; *P* =0.010) at the follow-up endpoint. Substantial statistical heterogeneity exists among the studies (I^2^ = 90.0 %; *P* <0.001).

**Table 2:** Pooled estimates for IOPR from baseline for Ahmed versus Baerveldt

	No. of studies	WMD(random)(95 % CI)	Test for heterogeneity	Test for overall effect
6 months				
All trials	5	1.58 (−2.99, 6.15)	Q =83.02, *P* <0.001	Z =0.68, *P* =0.499
Retro	3	2.52 (−4.81, 9.84)	Q =31.53, *P* <0.001	Z =0.67, *P* =0.500
RCT	2	0.06 (−1.34, 1.45)	Q =0.83, *P* =0.362	Z =0.08, *P* =0.937
12 months				
All trials	6	−1.01 (−3.40, 1.98)	Q =35.80, *P* <0.001	Z =0.66, *P* =0.509
Retro	4	0.29 (−3.12,3.70)	Q =12.12, *P* =0.007	Z =0.17, *P* =0.869
RCT	2	−3.16 (−4.86,−1.45)	Q =0.16, *P* =0.687	Z =3.63, *P* <0.001
24 months				
All trials	3	−0.54 (−4.89, 3.82)	Q = 21.78, *P* <0.001	Z =0.24, *P* =0.809
Retro	2	0.28 (−5.68, 6.24)	Q = 10.11, *P* =0.001	Z = 0.09, *P* =0.926
RCT	1	−2.27 (−4.68, 0.14)	-	Z =1.85, *P* =0.065
36 months				
All trials	2	−0.47 (−3.29, 2.35)	Q = 4.44, *P* =0.035	Z = 0.32, *P* =0.746
Retro	1	0.80 (−0.46, 2.06)	-	Z = 1.24, *P* =0.215
RCT	1	−2.10 (−4.48, 0.28)	-	Z = 1.73, *P* =0.084

*IOPR* intraocular pressure reduction, *WMD* weighted mean differences, *CI* confidence interval, *Retro* retrospective comparative study, *RCT* prospective randomized controlled trial

### Success rate

**Fig. 2:**
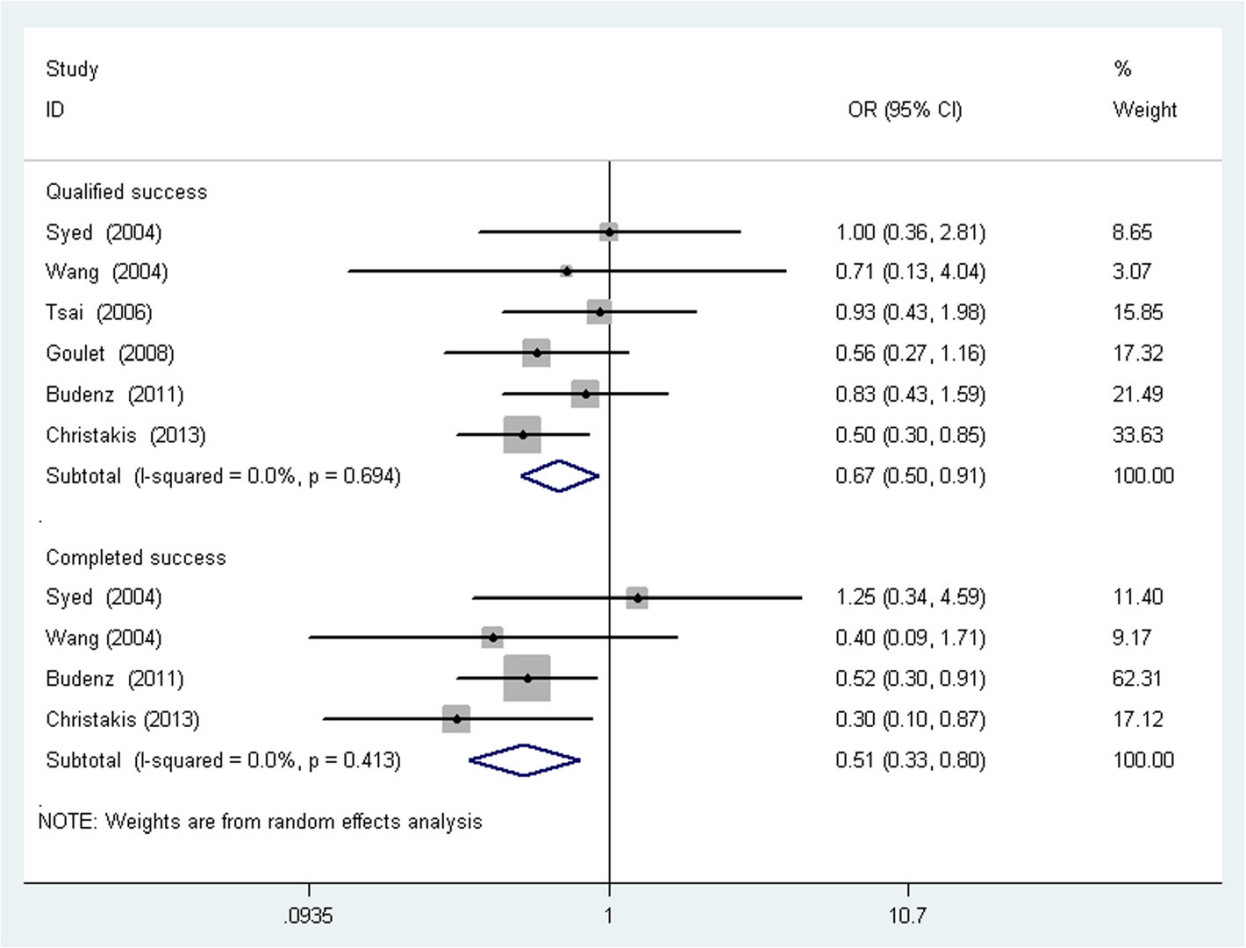
Forest figure of success rate comparing the AGV implant with the Baerveldt implant. (Odd ratio (OR) were computed using a random effects model. Ninety-five percent CI indicates 95 % confidence interval)

All six studies reported the proportions of patients that achieved the target endpoint IOP with or without medication at the follow-up end point. The qualified success rate of the Baerveldt implant is significantly higher than that of the AGV implant (pooled OR: 0.67, 0.50 to 0.91; *P* =0.011) (Fig. 2). Four studies reported the complete success rate at the follow-up endpoint. The pooled result indicated that the complete success rate of the Baerveldt implant is also significantly higher than that of the AGV implant (pooled OR: 0.51, 0.33 to 0.80; *P* =0.003). No substantial statistical heterogeneity was observed between studies (Fig. 2).

### Side effects

The results of the analysis of postoperative complications are shown in Table 3. The incidence of hyphema (pooled OR: 0.62, 0.35 to 1.12), flat anterior chamber (pooled OR: 0.89, 0.56 to 1.39), hypotony (pooled OR: 1.10, 0.52 to 2.37), choroidal effusion (pooled OR: 1.15, 0.66 to 1.98), suprachoroidal hemorrhage (pooled OR: 0.64, 0.21 to 1.99), retinal detachment (pooled OR: 0.43, 0.10 to 1.76), endophthalmitis (pooled OR: 1.35, 0.24 to 7.67), tube blockage (pooled OR: 0.85, 0.04 to 16.63), and tube exposure (pooled OR: 0.66, 0.09 to 5.18) were comparable in Ahmed implants and Baerveld implants.

**Table 3:** Adverse events from Ahmed and Baerveldt compared

	No. of studies	Crude Rate, n/N (%)		OR (95 % CI)			Test for Heterogeneity			Test for Overall Effect	
Adverse event		Ahmed	Baerveldt	Estimate	Lower	Up	*x* ^2^	I^2^	*P*	Z	*P*
Hyphema	4	21/317	31/303	0.62	0.35	1.12	2.10	0.00 %	0.551	1.57	0.116
Flat anterior chamber	3	45/285	47/271	0.89	0.56	1.39	0.75	0.00 %	0.688	0.53	0.596
Hypotony	4	18/282	18/368	1.10	0.52	2.37	1.26	0.00 %	0.738	0.26	0.797
Choroidal effusion	4	40/333	35/341	1.15	0.66	1.98	3.29	8.90 %	0.349	0.49	0.625
Suprachoroidal hemorrhage	5	4/406	12/482	0.64	0.21	1.99	4.11	2.70 %	0.391	0.77	0.440
Retinal detachment	3	2/231	8/317	0.43	0.10	1.76	0.15	0.00 %	0.928	1.18	0.238
Endophthalmitis	3	3/315	2/317	1.35	0.24	7.67	1.46	0.00 %	0.483	0.34	0.732
Tube blockage	2	6/161	13/157	0.85	0.04	16.63	4.99	79.90 %	0.026	0.11	0.912
Tube exposure	2	1/202	3/266	0.66	0.09	5.18	0.12	0.00 %	0.724	0.39	0.696

*OR* odds ratio, *CI* confidence interval

### Publication bias

Begg’s rank correlation test (*P* = 0.452) and Egger’s linear regression test (*P* = 0.236) were based on IOP reduction at 12 months and indicated no obvious publication bias for any of the parameters.

## Discussion

In the present meta-analysis, existing literature has been reviewed regarding the efficacy and safety of the AGV implant and the Baerveldt implant in the treatment of refractory glaucoma. The pooled results of this meta-analysis suggest that the Baerveldt implant archive a greater number of glaucoma medications reduction than the AGV implant, and had a relative higher completed and qualified success rate than the AGV implant. This indicates that the Baerveldt implant is more effective than the AGV implant in the management of refractory glaucoma. Findings indicate that the Baerveldt and Ahmed implants are comparable in terms of IOPR outcomes.

Glaucoma drainage implants have been in use for more than two decades; different drainage implants might affect the efficacy of glaucoma surgery for refractory glaucoma. The present meta-analysis found no statistically significant differencein the IOPR between Baerveldt and AGV implants during the intervals. Subgroup analysis also did not materially alter the IOPR pooled results.

This meta-analysis also demonstrated that there are significant differences between the two implants with respect to some important clinical outcomes, including the number of glaucoma medications administered and the success rate. While these outcomes are not always the most important or the primary outcomes, these differences should not be ignored. The exact mechanisms through which the Baerveldt implant achieves a higher success rate than the AGV implant remains unclear. This difference may be (partially) accounted for by two explanations. First, the Baerveldt implant’s larger surface area might yield better efficacy at lowering IOP. Second, the lack of valve-induced resistance in the Baerveldt implant might more easily promote the aqueous humor flow to the plate.

The overall postoperative complication rates were comparable between the two implants. The AGV implant has a restrictive “valve” device designed to prevent hypotony. The Baerveldt implant does not have a flow restrictor; therefore, hypotony and its resultant complications would theoretically be much more common [29]. However, this meta-analysis indicated that Baerveldt implants did not differ with AGV implants with respect to hypotony complications. In fact, in clinical practice, Baerveldt implants were always restricted by the surgeon using a suture ligature; this may be an important factor for decreasing this common complication. Unfortunately, some of the studies did not report overall complications but only postoperative complications leading to surgical failures; these omissions may have introduced bias [13, 18].

In the present meta-analysis, substantial statistical heterogeneity was not found among studies for most dichotomous outcomes but was found for the majority of continuous variables. Concerning this heterogeneity, several causes might be the main reason: First, there are different inclusion criteria and different operative techniques in the different studies. Next, different surgeons’ experience, different surgical indications, and different outcome measurements might also have introduced potential bias. In order to reduce the effect of heterogeneity, we adopted the random-effects model to pool the data. However, this will not abolish the heterogeneity completely.

This study had a number of strengths. First, it presents a direct, rather than indirect, comparison between the AGV implant and the Baerveldt implant. Second, the present meta-analyses separated the studies by the length of follow-up when analyzing the IOPR outcome. Third, it strictly follows the *Cochrane Handbook for Systematic Reviews of Interventions* and the PRISMA Statement. This ensures that the conclusions are more stable, reliable, and scientific. Thus, this meta-analysis provides the most up-to-date information in this area.

However, some limitations in this meta-analysis should not be ignored. First, in reviewing the literature, only a few randomized studies were included. Second, heterogeneity exists between studies. A random effects model was used in order to obtain a conservative estimate when statistically significant heterogeneity was met. Third, as we cannot attempt to gain access to unpublished results, publication bias cannot be fully excluded. Fourth, variables such as the success rate, the number of glaucoma medications, and the tolerability estimates were all pooled from trials of different durations because of a lack of reported data in all follow-up phases. It was a compromise proposal to choose the data of follow-up end point. Fifth, different studies adopted different criteria for a normal IOP. It might be another source of heterogeneity in the results was the assessment criterion for success. In addition, all of the studies that were included had follow-up periods until 12 months, after which the number of studies included reduced (3 at 24 months and 2 at 36 months). A glaucoma drainage device generally remains functional after only 1 year; it is doubtful if the differences between the two devices are meaningful. Thus, future studies with extensive follow-ups would be included in the meta-analysis. Finally, the majority of included subjects were Caucasian; only one study, consisting of 24 eyes, was from Singapore.

## Conclusion

In summary, this meta-analysis suggests primarily that the Baerveldt and Ahmed implants are comparable in IOP reduction outcomes. A second conclusion may be that the Baerveldt implant is more effective both in its surgical success rate and in its reduction of glaucoma medication. However, we should consider the inherent limitations of this meta-analysis, and conclusions should be interpreted with caution. Future well-designed RCTs with extensive follow-ups should be performed to confirm and update these findings.

## Abbreviations

AGV, Ahmed glaucoma valve; CI, confidence interval; IOP, intraocular pressure; IOPR, intraocular pressure reduction; OR, odds ratio; RCT, randomized clinical trial; SD, standard deviations; WMD, weighted mean difference
